# The Effects of Multimodal Prehabilitation on Postoperative Outcomes in Colorectal Surgery: A Systematic Review and Meta-Analysis

**DOI:** 10.7759/cureus.95032

**Published:** 2025-10-21

**Authors:** Nirmani Widanage, Ahmed Almonib, Kasun Gunathilaka

**Affiliations:** 1 General and Colorectal Surgery, George Eliot Hospital NHS Trust, Nuneaton, GBR; 2 General Surgery, Queen Elizabeth Hospital Birmingham, Birmingham, GBR; 3 General Surgery, National Cancer Institute Sri Lanka, Maharagama, LKA

**Keywords:** 6-minute walk test, colorectal cancer, colorectal resections, enhanced recovery after surgery (eras) protocols, multimodal prehabilitation, systematic review and meta analysis

## Abstract

Colorectal surgery carries risks of functional decline and complications. Multimodal prehabilitation, integrating exercise, nutrition, and psychological support, may enhance recovery alongside Enhanced Recovery After Surgery (ERAS) pathways. This review evaluated its impact on postoperative outcomes.

A systematic review and meta-analysis were conducted using a standardized and transparent methodology to ensure comprehensive and unbiased reporting. PubMed, Embase (via Ovid), MEDLINE (via Ovid), and Cochrane Central were searched for randomized controlled trials (RCTs). Eligible trials compared multimodal prehabilitation with standard care in elective colorectal resections. The primary outcome was overall postoperative complications; secondary outcomes included functional capacity by the six-minute walk test (6MWT) and severe complications (Clavien-Dindo ≥III). Five RCTs (422 patients) were included. Prehabilitation significantly lowered overall postoperative complications compared with standard of care (OR: 0.60, 95% CI: 0.42-0.86; p=0.02; I²=0%). Functional recovery was improved, with a pooled mean 6MWT difference at eight weeks of +50.8 m (95% CI: 25.9-75.7; p<0.0001; I²=59%). No significant reduction was observed for severe complications (OR: 0.65, 95% CI: 0.13-3.29; p=0.37; I²=20%). Multimodal prehabilitation improves functional recovery and reduces overall complications in colorectal cancer surgery, though its effect on severe complications remains uncertain. Larger multicenter trials are needed to strengthen the evidence base.

## Introduction and background

Colorectal surgery is among the most commonly performed major abdominal procedures worldwide. Colorectal resections are commonly performed due to colorectal cancer, but also for conditions such as diverticular disease, inflammatory bowel disease, and benign polyps. In most centers, operations for colon cancer are more common than for rectal cancer, though both carry similar perioperative risks. Despite advances in minimally invasive techniques and the widespread implementation of Enhanced Recovery After Surgery (ERAS) pathways, patients undergoing colorectal resections remain at risk of postoperative complications, delayed recovery, and functional decline, which may adversely affect long-term outcomes and healthcare utilization [1]. These limitations underscore the need for additional perioperative strategies to optimize recovery further.

Prehabilitation has emerged as a proactive approach designed to enhance patients’ physiological and psychological reserve before surgery. Unlike rehabilitation, which is delivered postoperatively, prehabilitation targets the preoperative period to improve resilience against surgical stress [2]. Multimodal programs commonly incorporate structured exercise training, nutritional optimization, and psychological support, with the potential for synergistic effects on functional capacity and postoperative outcomes.

Although several randomized trials and recent reviews suggest that prehabilitation may improve recovery, findings remain inconsistent, and its role within modern ERAS pathways for colorectal resections has not been firmly established. This systematic review and meta-analysis synthesize randomized evidence to evaluate the effectiveness of multimodal prehabilitation on postoperative outcomes in patients undergoing colorectal surgery.

This systematic review and meta-analysis was previously presented as an oral presentation on September 16, 2025, at the Sixth Edition of Global Conference on Surgery and Anesthesia 2025, London, United Kingdom.

## Review

Materials and methods

This review was designed and reported in line with the Preferred Reporting Items for Systematic Reviews and Meta-Analysis (PRISMA) 2020 framework [3]. No protocol for this review was registered in the International Prospective Register of Systematic Reviews (PROSPERO) or other registries of systematic reviews.

We included randomized controlled trials evaluating multimodal prehabilitation in patients undergoing elective colorectal surgery that reported at least one prespecified outcome - overall postoperative complications; major complications (defined as Clavien-Dindo grade ≥III or a comprehensive complication index {CCI} >20, both of which are publicly available grading and scoring systems); or functional recovery, as measured by the six-minute walk test (6MWT) [4,5].

Eligibility was defined using the PICO framework, i.e., Patients - adults (>18 years) undergoing colorectal surgery; Intervention - multimodal prehabilitation (≥2 components such as exercise, nutrition, or psychological support); Comparator - standard perioperative care with or without ERAS; Outcomes - postoperative complications and functional recovery. Only full-text studies published in English were considered.

We excluded study protocols, reviews, observational or non-randomized studies, conference abstracts, non-English reports, trials involving non-colorectal populations, single-modality interventions, or outcomes outside the scope of this review.

Search Strategy

We systematically searched PubMed, MEDLINE via Ovid, Embase via Ovid, and the Cochrane Central Register of Controlled Trials (CENTRAL) for publications appearing between January 1, 2015, and August 1, 2025. Search strategies combined controlled vocabulary and free-text terms related to “prehabilitation,” “multimodal prehabilitation,” “colorectal surgery,” “anastomotic leak,” “postoperative complications,” and “six-minute walk test (6MWT),” with Boolean operators, and were then applied appropriately.

All retrieved records were imported into Rayyan software (Cambridge, MA: Rayyan Systems Inc.), a free web-based application designed for systematic reviews for management [6]. Duplicates were removed before screening. Two reviewers (NW and AA) independently screened the titles and abstracts, followed by a full-text review of potentially eligible studies. Any disagreements were resolved after consultation with a third reviewer (KG).

Data Extraction

Data from the included studies were extracted independently by two reviewers (NW and AA) using a predefined standardized form. The following information was collected: study characteristics (author, year, country, sample size, and study design), patient demographics, details of the multimodal prehabilitation intervention and control group, reported outcomes, and key findings. Outcomes of interest included overall postoperative complications, serious complications (Clavien-Dindo ≥III, CCI >20), and functional capacity measured by the six-minute walk test (6MWT). Any discrepancies in data extraction were resolved by consensus or, if required, discussion with a third reviewer.

Risk of Bias Assessment

The methodological quality of each included study was assessed using the Cochrane Risk of Bias 2.0 tool, which is freely available from the Cochrane Collaboration (Copenhagen, Denmark) [7]. Each domain was rated as “low risk,” “some concerns,” or “high risk,” and an overall risk of bias was assigned accordingly.

Data Synthesis and Statistical Analysis

Meta-analysis was conducted using Review Manager (RevMan version 5.4, Copenhagen, Denmark: Cochrane Collaboration), which is freely available for researchers [8]. For dichotomous outcomes (overall and severe postoperative complications), pooled odds ratios (OR) with 95% confidence intervals (CI) were calculated. For continuous outcomes (six-minute walk test distance), mean differences (MD) with 95% CI were used. A random-effects model was applied to account for expected clinical and methodological heterogeneity. Statistical heterogeneity was evaluated using the I² statistic, with values >50% considered substantial. Sensitivity analyses excluding studies at high risk of bias were planned to assess the robustness of findings.

Certainty of Evidence

The certainty of evidence for the main outcomes (functional capacity measured by the 6MWT, overall postoperative complications, and severe complications) was evaluated using the Grading of Recommendations Assessment, Development and Evaluation (GRADE) approach, which is freely available through the GRADE working group [9]. Certainty was assessed across the following five domains: risk of bias, inconsistency, indirectness, imprecision, and publication bias.

Results

**Figure 1:**
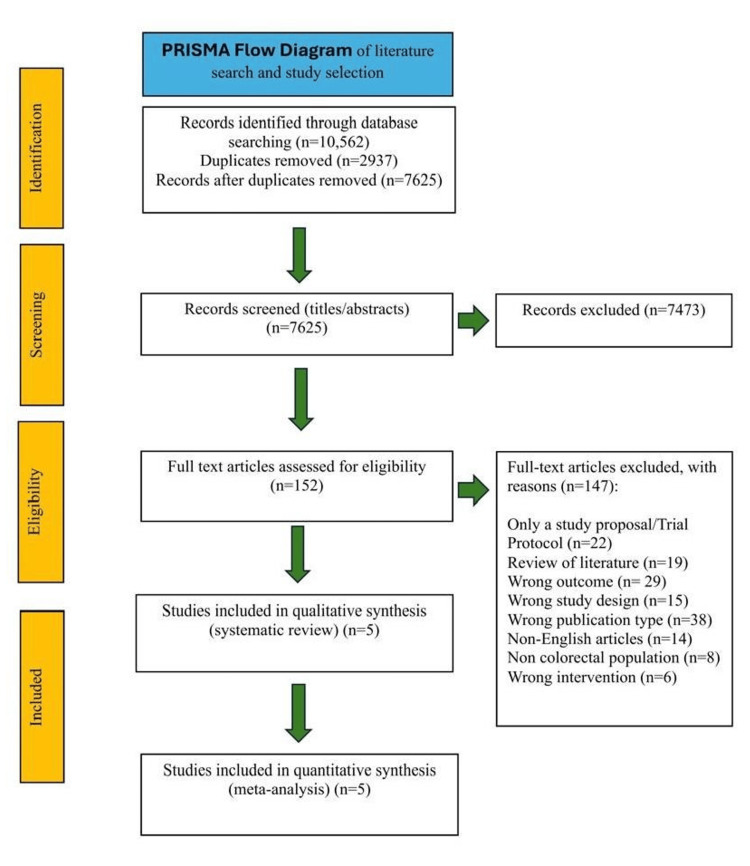
PRISMA 2020 flow diagram illustrating the process of literature search, screening, eligibility assessment, and final study inclusion. PRISMA: Preferred Reporting Items for Systematic Reviews and Meta-Analysis; n: number of records

The initial search retrieved 10,562 records - PubMed (n=1,909), MEDLINE via Ovid (n=891), Embase via Ovid (n=6,048), and the Cochrane Central Register of Controlled Trials (n=1,714). All retrieved records were imported into the Rayyan software for management. After removal of 2,937 duplicates, 7,625 records remained for title and abstract screening. Of these, 7,473 were excluded, leaving 152 full-text articles for eligibility assessment. A further 147 were excluded for the following reasons: study protocol/proposal (n=22), review article (n=19), wrong outcome (n=29), wrong study design (n=15), wrong publication type (n=38), non-English article (n=14), non-colorectal population (n=8), and wrong intervention (n=6). Ultimately, five randomized controlled trials met the inclusion criteria and were included in both the qualitative (systematic review) and quantitative (meta-analysis) analyses [10-14]. The study selection process is summarized in the PRISMA 2020 flow diagram (Figure 1) [3].

Characteristics of the Included Studies

Five randomized controlled trials were included, enrolling a total of 422 patients undergoing elective colorectal cancer surgery. Sample sizes ranged from 20 to 251 participants. Interventions involved multimodal prehabilitation, typically combining physical exercise, nutritional support, and psychological or relaxation components, delivered either in-hospital or at home. Control groups received standard perioperative care without structured prehabilitation. All included studies evaluated patients undergoing elective colorectal cancer surgery (malignant disease). None involved benign indications. Reported outcomes included functional capacity (6MWT), postoperative complications, severe complications (Clavien-Dindo ≥III, CCI >20), and length of hospital stay [10-14]. Key characteristics of the included trials are summarized in Table 1.

**Table 1:** Study characteristics of the included trials. Study characteristics of randomized controlled trials evaluating multimodal prehabilitation in elective colorectal surgery. CPET: cardiopulmonary exercise testing; VO₂: oxygen consumption; LOS: length of hospital stay; CCI: comprehensive complication index; ERAS: enhanced recovery after surgery; 6MWT: six-minute walk test

Studies	Country/setting	Study design	Population	Intervention	Comparator	Outcomes measured	Key results
Pesce et al. (2024) [10]	Italy (single center - Ferrara)	Randomized controlled trial (interim n=71)	Adults scheduled for elective colorectal cancer surgery (n=35/36)	4-week trimodal prehabilitation: CPET-guided exercise, protein supplements, psychological support	No formal prehabilitation; ERAS-based care [1]	6MWT, VO₂ peak, handgrip, sit‑to‑stand, complications, LOS	Prehab group walked ~90-100 m farther at 4 and 8 weeks; complications and hospital stay were similar between groups
Molenaar et al. (2023) [11]	International multicenter trial	Randomized clinical trial (n=251)	Adults with non-metastatic colorectal cancer, elective surgery (n=123/128)	4-week supervised in-hospital multimodal prehabilitation: exercise, nutrition, psychological support, smoking cessation	Standard ERAS perioperative care	CCI score [5], severe complications (CCI >20), medical complications, 6MWT at 4 weeks postoperative	Fewer severe complications (17.1% vs. 29.7%, p=0.02); fewer medical complications (15.4% vs. 27.3%, p=0.02); 6MWT improvement not statistically significant
López-Rodríguez-Arias et al. (2021) [13]	Spain	Randomized controlled trial (n=20)	Patients undergoing elective colorectal cancer surgery (n=10/10)	Home-based multimodal prehabilitation: aerobic + resistance exercise, nutritional support, relaxation techniques	Standard ERAS care, no structured prehabilitation	6MWT, postoperative complications, hospital length of stay	Improved functional outcomes and lean body mass preservation; no significant difference in complications or LOS
Triguero-Cánovas et al. (2023) [14]	Spain	Randomized controlled trial (n=44)	Patients treated surgically for colorectal cancer (n=23/21)	Physical activity, nutritional supplementation, and relaxation exercises (home-based multimodal)	Usual care	6MWT, LOS, complications	Improved physical condition and 6MWT; effects on LOS and complications were not statistically significant
Bojesen et al. (2023) [12]	Denmark	Randomized controlled trial (n=36)	Patients with colorectal cancer surgery (n=16/20)	Physical activity, nutritional supplementation, and medical optimisation (hospital-based multimodal)	Usual care	6MWT, LOS, complications	Improved 6MWT; no significant difference in LOS or overall complications

**Figure 2:**
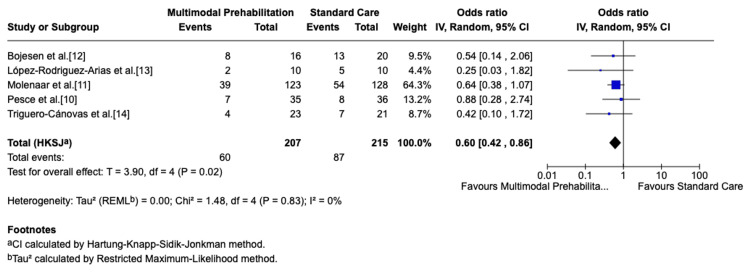
Forest plot of overall postoperative complications. Forest plot showing overall postoperative complications in patients undergoing multimodal prehabilitation compared with standard perioperative care. Effect sizes are presented as odds ratios (OR) with 95% confidence intervals (CI), using a random-effects model. IV: inverse variance; CI: confidence interval; HKSJ: Hartung-Knapp-Sidik-Jonkman; REML: Restricted Maximum Likelihood

Risk of bias was judged using the RoB 2 tool. Two trials were at low risk [10,11], while three were judged as having some concerns [12-14]. The results of the RoB 2 assessment are presented in Table 2. Five randomized trials (n=422) reported data on overall postoperative complications. Multimodal prehabilitation significantly reduced the risk of complications compared to standard care (OR: 0.60; 95% CI: 0.42-0.86; p=0.02). Statistical heterogeneity was low (I²=0%), suggesting consistency of findings across trials. These results are illustrated in the forest plot (Figure 2) [10-14].

**Table 2:** Risk of bias (RoB 2) assessment at the study level for postoperative complications. Risk of bias was judged using the RoB 2 tool at the study level for postoperative complications [7]. Judgments were classified as low, some concerns (SC), or high across the following five domains: domain 1 - randomization process; domain 2 - deviations from intended interventions (effect of assignment); domain 3 - missing outcome data; domain 4 - outcome measurement; and domain 5 - selection of the reported result. Overall risk of bias was rated low only if all domains were low; high if any domain was high; otherwise, some concerns (SC).

Studies	Domain 1	Domain 2	Domain 3	Domain 4	Domain 5	Overall (ROB)
Molenaar et al. (2023) [11]	Low	Low	Low	Low	Low	Low
Bojesen et al. (2023) [12]	SC	SC	Low	Low	Low	SC
López-Rodríguez-Arias et al. (2021) [13]	SC	Low	Low	Low	Low	SC
Pesce et al. (2024) [10]	Low	Low	Low	Low	Low	Low
Triguero-Cánovas et al. (2023) [14]	SC	SC	SC	Low	Low	SC

**Figure 3:**
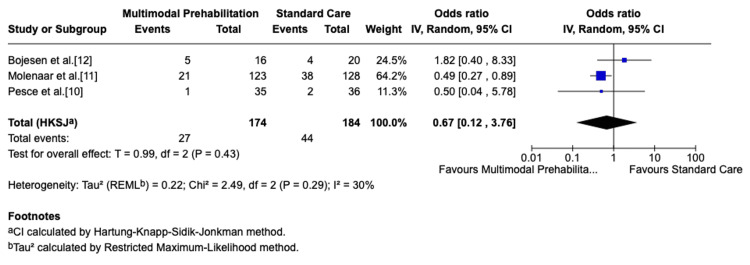
Forest plot of severe postoperative complications. Forest plot showing severe postoperative complications in patients who underwent multimodal prehabilitation compared with standard perioperative care. Effect sizes are presented as odds ratios (OR) with 95% confidence intervals (CI), using a random-effects model. IV: inverse variance; CI: confidence interval; HKSJ: Hartung-Knapp-Sidik-Jonkman; REML: Restricted Maximum Likelihood

Three trials (n=358) assessed severe complications, defined as Clavien-Dindo grade ≥III. Pooled analysis favored prehabilitation, but the reduction was not statistically significant (OR: 0.65; 95% CI: 0.13-3.29; p=0.37). Heterogeneity was low (I²=20%), indicating consistent results across studies. These results are illustrated in the forest plot (Figure 3) [10-12].

**Figure 4:**
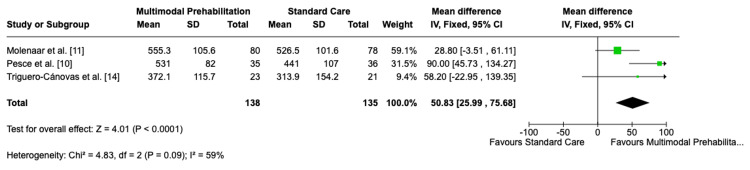
Forest plot of functional capacity. Forest plot showing functional capacity measured by 6MWT at eight weeks postoperatively in patients undergoing multimodal prehabilitation compared with standard perioperative care. Effect sizes are presented as mean difference (MD) with 95% CI, using a random-effects model. IV: inverse variance; CI: confidence interval; 6MWT: six-minute walk test

Three trials (n=273) reported 6MWT outcomes at eight weeks postoperatively. Multimodal prehabilitation resulted in a significant improvement in walking distance compared with standard care (MD: 50.8 m; 95% CI: 25.9-75.7; p<0.0001). Moderate heterogeneity was observed (I²=59%), reflecting variability in effect size between studies. These results are illustrated in the forest plot (Figure 4) [10,11,14].

Certainty of evidence, evaluated using the GRADE framework, was rated low for 6MWT due to risk of bias, inconsistency, and imprecision. Overall complications and severe complications were both judged to be of moderate certainty, supported by consistent findings and the large, low-risk Prehabilitation in Elective Patients Undergoing Resection of Colorectal Cancer (PREHAB) trial [11]. Certainty of evidence ratings according to the GRADE framework are summarized in Table 3.

**Table 3:** GRADE evidence profile. GRADE evidence profile for the main outcomes was 6MWT, overall postoperative complications, and severe complications. Certainty of evidence was assessed across the following five GRADE domains [9]: study limitations (risk of bias), inconsistency, imprecision, indirectness, and publication bias. Quality of evidence was graded as high, moderate, low, or very low. Here, high grade = very high confidence in the effect estimate; moderate = moderately confident; low = limited confidence; very low = very little confidence. The symbol "↓" represents a one-level downgrade for the domain, and the symbol "-" represents no serious limitation. GRADE: Grading of Recommendations, Assessment, Development, and Evaluations; 6MWT: six-minute walk test; RoB: risk of bias; RCT: randomized controlled trial; SC: severe complications; PREHAB: Prehabilitation in Elective Patients Undergoing Resection of Colorectal Cancer

Outcomes	No. of studies (type)	RR (95% CI)	Mean difference (95% CI)	Limitations in study design	Inconsistency	Imprecision	Quality of evidence
6MWT (functional capacity)	3-4 RCTs	NA	≈ +30-100 m (wide CI)	↓ (Risk of bias, attrition, performance bias)	↓ (Heterogeneity in effect size)	↓ (Underpowered, CI not always reported)	Low
Overall postoperative complications	4-5 RCTs	0.8-1.0 (CI crosses 1)	NA	↓ (Mix of low, SC RoB)	- (Results consistent)	- (Large PREHAB trial precise)	Moderate
Severe complications (Clavien-Dindo ≥III/CCI >20) [4,5]	2-3 RCTs	≈0.47 (0.26-0.87)	NA	↓ (Only one large low RoB RCT, others small)	- (Results consistent)	- (Large PREHAB trial precise)	Moderate

Discussion

This systematic review and meta-analysis evaluated the impact of multimodal prehabilitation on functional capacity and postoperative outcomes in colorectal cancer surgery. Prehabilitation significantly improved postoperative 6MWT distance and reduced the risk of overall complications when compared to the standard of care. Although fewer severe complications were observed, the pooled effect did not reach statistical significance.

The observed improvement in functional recovery is consistent with earlier trials and reviews, demonstrating that prehabilitation can mitigate the decline in physical capacity associated with major abdominal surgery. The pooled increase of around 50 m in 6MWT at eight weeks is clinically relevant, as even modest improvements in functional reserve have been associated with better postoperative recovery and quality of life. Moderate heterogeneity was identified, likely due to differences in exercise intensity, supervision, adherence, and patient selection, which underscores the need for more standardized and scalable intervention protocols.

The PREHAB trial further strengthens the evidence base by providing prospective randomized data on multimodal prehabilitation. This trial demonstrated that patients undergoing colorectal surgery who received a structured program combining exercise, nutritional counselling, and psychological support achieved significant improvements in functional capacity prior to surgery and experienced fewer postoperative complications compared with standard of care. Importantly, the trial highlighted that benefits were most pronounced in patients at higher baseline risk, suggesting that prehabilitation may be particularly impactful in vulnerable populations. In addition, adherence to the program was high, underscoring the feasibility of delivering multimodal interventions in a perioperative setting. These findings align with the results of recent systematic reviews and meta-analyses, reinforcing the concept that well-structured, multimodal prehabilitation can meaningfully influence both short-term recovery and longer-term outcomes.

Similar findings were reported in recent systematic reviews, including Zhou et al., Steffens et al., and Liao et al., which collectively support the role of prehabilitation in improving postoperative outcomes [15-17]. Recent literature continues to support the potential benefits of prehabilitation in patients undergoing colorectal surgery, though findings highlight ongoing challenges and nuances. Zhang et al. demonstrated in their systematic review and meta-analysis that prehabilitation significantly improves postoperative outcomes, particularly by reducing complication rates and enhancing recovery trajectories [18]. Similarly, Sabajo et al. emphasized that prehabilitation programs not only improve clinical outcomes but may also contribute to reducing hospital costs, underlining the broader healthcare value of such interventions [19]. However, translating these findings into real-world practice is complex. Talen et al. highlighted the difficulties of implementing evidence-based prehabilitation within routine clinical pathways, noting variability in program adherence, patient engagement, and logistical feasibility [20]. These practical limitations may partially explain the heterogeneity seen across studies and should be considered when interpreting pooled results.

From a functional outcomes perspective, Maroto-Izquierdo et al. provided robust evidence through their systematic review and meta-analysis that concurrent exercise-based prehabilitation significantly enhances physical performance, particularly six-minute walk distance, underscoring its role in optimizing functional reserve [21]. Finally, Sethi et al. broadened the discussion by showing that nutritional prehabilitation, alone or combined with exercise, yields favorable effects on postoperative outcomes, reinforcing the importance of a multimodal approach [22].

The reduction in overall complications reinforces the potential value of prehabilitation within modern ERAS pathways. Even when patients already benefit from ERAS, adding structured rehabilitation appears to confer additional protection against postoperative morbidity. Complications drive longer hospital stays, higher costs, and delays in adjuvant therapy, making these findings clinically important. Importantly, the direction of effect was consistent across trials, with no study showing harm. These results align with prior evidence syntheses that have also demonstrated a consistent trend toward reduced morbidity following multimodal prehabilitation.

For severe complications, the largest and most robust trial, the PREHAB trial, reported a clear benefit, but this effect was diluted in pooled analysis by smaller, underpowered studies. As such, the estimate suggested a possible reduction but without statistical significance. This highlights the need for further adequately powered multicenter trials to determine whether prehabilitation can reliably lower high-grade complication rates.

Strengths and Limitations

This review provides the most up-to-date synthesis of randomized evidence (to August 2025) on multimodal prehabilitation in colorectal surgery. Unlike previous reviews, it focuses exclusively on multimodal interventions and excludes single-modality approaches. Only randomized controlled trials were included, ensuring a higher-quality evidence base. The review adhered to the PRISMA 2020 guidelines and applied rigorous methodology, including duplicate screening, data extraction, and independent risk of bias assessment (RoB2). Certainty of evidence was evaluated using the GRADE approach, enhancing transparency in interpretation.

The review was not prospectively registered in PROSPERO, which may affect methodological transparency. The number of eligible trials was small, many were single-center studies with modest sample sizes, and intervention protocols varied, contributing to heterogeneity. Risk of bias assessment revealed concerns in several trials, and the GRADE evaluation rated the certainty of evidence as low to moderate. For the PREHAB trial, standard deviations had to be derived from available data due to some missing data, which may have introduced minor estimation errors. Publication bias could not be formally assessed due to the limited number of included studies.

## Conclusions

This systematic review and meta-analysis demonstrates that multimodal prehabilitation before elective colorectal surgery is associated with improved functional capacity and a reduction in overall postoperative complications compared to standard of care. Evidence for a reduction in severe complications remains less certain but suggests a potential benefit. Enhancing functional capacity before surgery may increase resilience, reduce morbidity, and enable earlier initiation of adjuvant therapy. Most programs were multimodal, integrating exercise, nutrition, and psychological support, which likely act synergistically. However, implementation requires consideration of resource allocation and adherence. Home-based models, used during the COVID-19 pandemic, may offer practical solutions if accompanied by adequate support and monitoring.

These findings suggest that prehabilitation should be considered as an adjunct to ERAS care in colorectal surgery, while further large multicenter trials are needed to confirm its impact on high-grade complications and to establish standardized protocols.
